# Effects of Obstructive Sleep Apnea Hypopnea Syndrome on Blood Pressure and C-Reactive Protein in Male Hypertension Patients

**DOI:** 10.14740/jocmr2409w

**Published:** 2016-01-26

**Authors:** Fan Li, Hui Huang, Ligong Song, Hua Hao, Mingzhong Ying

**Affiliations:** a The International Medical Center of the People’s Liberation Army General Hospital, District 3, Division 2, 28 Fuxin Road, Beijing 100853, China

**Keywords:** Obstructive sleep apnea hypopnea syndrome, Hypertension, C-reactive protein

## Abstract

**Background:**

The influences of obstructive sleep apnea hypopnea syndrome (OSAHS) on blood pressure and C-reactive protein (CRP) were observed, and the underlying mechanism was investigated.

**Methods:**

Respiratory sleep monitoring was performed on 188 male patients who were newly diagnosed with hypertension. Based on the apnea hypopnea index (AHI) results, patients were divided into a normal control group (AHI ≤ 5, n = 35), a mild OSAHS group (5 < AHI ≤ 15, n = 28), a moderate OSAHS group (15 < AHI ≤ 30, n = 57), and a severe OSAHS group (AHI > 30, n = 68). Ambulatory blood pressure monitoring was conducted on patients in each group, and blood samples were collected to detect indicators, including fasting blood glucose (FBG), triglyceride (TG), low-density lipoprotein-cholesterol (LDL-C), high-density lipoprotein-cholesterol (HDL-C), and high-sensitivity CRP (hs-CRP).

**Results:**

TG and hs-CRP in patients in the moderate and severe OSAHS groups were higher than those in the normal control group (P < 0.01, P < 0.05). Additionally, their mean nocturnal systolic blood pressure (nSBP) and nocturnal diastolic blood pressure (nDBP) were higher than those in the normal control group (P < 0.01, P < 0.05). However, the percentage of blood pressure reduction at night was significantly lower than that in the normal control group (P < 0.01). AHI and hs-CRP positively correlated with nSBP (adjusted R^2^ = 0.46) and nDBP (adjusted R^2^ = 0.38) and negatively correlated with the nocturnal blood pressure reduction percentage (adjusted R^2^ = 0.48).

**Conclusion:**

Moderate and severe OSAHS induced increases in nocturnal blood pressure and CRP content in the body, resulting in further damage to the circadian rhythms of blood pressure.

## Introduction

Hypertension is one of the most important risk factors for patients with cardiovascular and cerebrovascular diseases. Achieving blood pressure control within the standard range has important significance for the reduction of cardiovascular mortality. However, studies have shown that when hypertension patients also had obstructive sleep apnea hypopnea syndrome (OSAHS), their blood pressure fluctuations were more obvious than those in general hypertension patients, and they had difficultly achieving control within the standard range [1]. Currently, it is known that hypoxemia caused by OSAHS influences blood pressure. However, are other factors involved in this regulation? As an inflammatory substance, C-reactive protein (CRP) is considered an important pathophysiological factor in the development and progression of hypertension. Therefore, this study performed respiratory sleep monitoring, ambulatory blood pressure monitoring, and laboratory tests on hypertension patients to investigate the correlations between OSAHS, blood pressure fluctuations, and CRP to guide clinical treatment.

## Patients and Methods

### Clinical data

A total of 188 male hypertension patients diagnosed and treated in our department from June 2014 to May 2015 who all conformed to the diagnostic criteria of the “2014 evidence-based guideline for the management of high blood pressure in adults” (JNC8) [2] were enrolled in this study. They had systolic blood pressure (SBP) ≥ 140 mm Hg and/or diastolic blood pressure (DBP) ≥ 90 mm Hg, they had clearly been diagnosed with hypertension, and they had not started anti-hypertensive drug treatment. Patients with mental disorders, significant upper respiratory tract obstructions, upper respiratory tract infections, diabetes mellitus, histories of cerebral hemorrhage or infarction, cardiac insufficiency (higher than grade III in the New York Heart Association (NYHA) Functional Classification), hypothyroidism, late-stage malignant tumors, and severe hepatic and renal dysfunction were excluded. The ages of the patients were between 38 and 62 years (mean 46 ± 7 years). The study protocol was approved by the Ethics Committee of the PLA General Hospital and all the patients provided written informed consent, according to the Declaration of Helsinki, prior to inclusion.

### Data collection

The patients’ ages, heights, and body weights were recorded. The body mass index (BMI) was calculated for each patient according to the formula: BMI = body weight (kg)/height^2^ (m^2^).

### Sleep monitoring and grouping

All study subjects received examinations using a portable sleep apnea recording device (ApneaLink^TM^, ResMed, Australia). On the first day of admission, the recording time was longer than 7 h. On the examination day, drinking alcohol and coffee and taking sedatives and sleeping pills were prohibited to avoid interfering with the assessments. The monitoring items included posture, nasal airflow, thoracoabdominal movement, and the minimum blood oxygen saturation (SpO_2_). On the next day, the sleep monitoring results of all patients were analyzed according to the 2003 Chinese OSAHS treatment guidelines (draft), of which the apnea hypopnea index (AHI) = ((number of apnea events + number of hypopnea events)/sleep time (min)) × 60. Based on the detected AHI values, the 188 patients were divided into 35 cases in the normal control group (AHI ≤ 5), 28 cases in the mild OSAHS group (5 < AHI ≤ 15), 57 cases in the moderate OSAHS group (15 < AHI ≤ 30), and 68 cases in the severe OSAHS group (AHI > 30).

### Ambulatory blood pressure monitoring

Starting from 8:00 - 9:00, 24-h ambulatory blood pressure monitoring using a portable type 90217 non-invasive cuff automatic blood pressure monitor (SPACELABS^TM^, USA) was conducted on all subjects. During the monitoring period, patients resumed their normal daily lives. The effective recording time of blood pressure monitoring had to be longer than 20 h. The effective reading of blood pressure > 85% of the time was regarded as qualified; otherwise, the readings had to be taken again. Computers automatically performed statistical analyses of ambulatory blood pressure monitoring data. The observation parameters included mean daytime systolic blood pressure (dSBP), mean daytime diastolic blood pressure (dDBP), mean nocturnal SBP (nSBP), and mean nocturnal DBP (nDBP). In addition, the nocturnal blood pressure reduction percentage was calculated using the formula (dSBP - nSBP)/dSBP × 100%, which represented the amplitudes of circadian blood pressure changes in patients.

### Laboratory examination

During the next morning, when receiving respiratory sleep monitoring (after fasting overnight for 12 h), patients’ cubital vein blood samples were collected to detect fasting blood glucose (FBG), triglyceride (TG), high-density lipoprotein-cholesterol (HDL-C), low-density lipoprotein-cholesterol (LDL-C), and high-sensitivity CRP (hs-CRP). FBG, TG, HDL-C and LDL-C were measured using the 7200 automatic biochemical analyzer (Japan), and hs-CRP was measured using an enzyme-linked immunosorbent assay (ELISA); the reagent kit was purchased from Shanghai Senxiong BioTechnology Co., Ltd. The detection procedure and calculation were performed strictly according to the instructional manual.

### Statistical methods

Statistical analysis was performed using the software SPSS version 17.0. Measurement data were presented as χ ± s. Comparison of mean values between groups was performed using the F test. Factors that affected blood pressure were obtained using a stepwise multiple linear regression analysis. P < 0.05 indicated that the difference was statistically significant.

## Results

### Comparison of clinical and biochemical data

According to the respiratory sleep monitoring results, the numbers of cases in each group were 35 cases in the normal control group, 28 cases in the mild OSAHS group, 57 cases in the moderate OSAHS group, and 68 cases in the severe OSAHS group. Compared to the normal control group, patients in the OSAHS groups had increased BMIs and TG and hs-CRP levels; of these measurements, the changes in patients in the moderate and severe groups were significantly different (P < 0.01, P < 0.05). Other indicators did not have changes (P > 0.05) (Table 1).

**Table 1:** Comparison of Clinical and Biochemical Data Among All Groups

Item	Normal control group (n = 35)	Mild OSAHS group (n = 28)	Moderate OSAHS group (n = 57)	Severe OSAHS group (n = 68)
Age (year)	43 ± 9	41 ± 12	45 ± 8	45 ± 7
BMI (kg/m^2^)	25.62 ± 2.79	26.07 ± 4.11	26.80 ± 2.54*	27.15 ± 2.20*
FBG (mmol/L)	5.07 ± 1.61	5.29 ± 1.78	5.54 ± 1.43	5.47 ± 1.26
TG (mmol/L)	1.82 ± 1.80	2.74 ± 2.02	2.98 ± 1.97†	2.94 ± 1.79†
LDL-C (mmol/L)	2.88 ± 1.35	3.15 ± 1.69	3.07 ± 1.41	3.21 ± 1.32
HDL-C (mmol/L)	1.14 ± 0.47	1.06 ± 0.81	0.98 ± 0.35	0.92 ± 0.29
hs-CRP (mg/L)	2.33 ± 2.82	3.68 ± 6.56	5.71 ± 8.17*	6.45 ± 7.25†

Compared with the normal control group, *P < 0.05 and †P < 0.01.

### Blood pressure changes

The dSBP and dDBP values of hypertension patients among the different groups were not significantly different (P > 0.05). However, the nSBP and nDBP values in patients in the three OSAHS groups were all higher than those in the normal control group. The nocturnal blood pressure reduction percentage significantly decreased; when the degree of OSAHS was more serious, the percentage of the reduction of nocturnal blood pressure was lower, and the difference between the moderate and severe OSAHS groups and the normal control group was statistically significant (P < 0.01). In addition, compared to the mild OSAHS group, the nSBP and nDBP values in patients in the moderate and severe OSAHS groups also significantly increased (P < 0.01, P < 0.05), and the nocturnal blood pressure reduction percentage significantly decreased (P < 0.01), while other indicators did not change (P > 0.05) (Table 2).

**Table 2:** Comparison of Mean Nocturnal Blood Pressure in Each Group

Group	Case number	dSBP (mm Hg)	dDBP (mm Hg)	nSBP (mm Hg)	nDBP (mm Hg)	Nocturnal blood pressure reduction percentage (%)
Normal control group	35	137.3 ± 14.6	88.1 ± 10.4	117.9 ± 11.2	77.4 ± 8.5	13.5 ± 5.4
Mild OSAHS group	28	135.4 ± 18.2	89.2 ± 12.1	119.1 ± 12.3	82.0 ± 11.4	11.8 ± 8.6
Moderate OSAH group	57	136.8 ± 13.9	90.4 ± 9.7	128.6 ± 17.1*,‡	86.7 ± 19.3*,†	5.8 ± 4.2*,‡
Severe OSAHS group	68	138.6 ± 11.5	91.7 ± 11.2	134.3 ± 16.4*,‡	90.3 ± 17.1*,‡	2.9 ± 2.1*,‡

Compared with the normal control group, *P < 0.01; compared with the mild OSAHS group, †P < 0.05 and ‡P < 0.01.

### Correlation analysis

The above examination results showed that the BMIs and the TG and hs-CRP levels of hypertension patients combined with OSAHS were all higher than those in general hypertension patients. In addition, the nocturnal blood pressure of hypertension patients combined with OSAHS was higher than that in general hypertension patients. Therefore, nSBP, nDBP, and the nocturnal blood pressure reduction percentage were used as dependent variables, and AHI, BMI, TG, and hs-CRP were used as independent variables to perform the stepwise multiple regression analysis to identify factors that affected nocturnal blood pressure. AHI and hs-CRP showed independent positive correlations with nSBP (adjusted R^2^ = 0.46) and nDBP (adjusted R^2^ = 0.38) and an independent negative correlation with the nocturnal blood pressure reduction percentage (adjusted R^2^ = 0.48) (Table 3).

**Table 3:** Analysis of Correlation Factors That Influenced Nocturnal Blood Pressure Changes in Patients

Dependent variable	Independent variable	Adjusted R^2^	P*	B	P†
nSBP	AHI	0.34	< 0.01	1.72	< 0.01
	hs-CPR	0.12	< 0.01	0.88	< 0.01
nDBP	AHI	0.29	< 0.01	1.64	< 0.01
	hs-CRP	0.09	< 0.01	0.83	< 0.01
Nocturnal blood pressure reduction percentage	AHI	0.30	< 0.01	-0.11	< 0.01
	hs-CRP	0.18	< 0.01	-0.35	< 0.01

*P value of the adjusted R^2^; †P value of B.

## Discussion

OSAHS is an airway obstruction and sleep apnea phenomenon with several causative factors. The main manifestations are daytime sleepiness and recurrent airway obstruction during sleep at night. OSAHS mainly occurs in obese and older people. More and more studies have discovered and confirmed that OSAHS is an independent risk factor for hypertension and is closely associated with hypertension [3]. Results in this study indicated that the influence of OSAHS on blood pressure mainly occurred at night. The nSBP and nDBP values of hypertension patients combined with OSAHS both increased to a certain extent. This influence was even more evident in patients with moderate to severe OSAHS. More and more evidence indicated that when the mean blood pressure level was the same or similar, hypertension patients with decreased or nonexistent circadian rhythms of blood pressure had significantly increased risks for the development of target organ injury and cardiovascular and cerebrovascular events; in addition, there is a vicious cycle between target organ injury and abnormal circadian rhythms of blood pressure [4]. Normal blood pressure displays a low in the nighttime and high in the daytime rhythm; in fact, the nighttime blood pressure is lower than that at daytime by at least 10%. During changes in the circadian rhythms of blood pressure, the mean blood pressure at night might be even higher than that at daytime. In this study, we found that hypertension patients with moderate to severe OSAHS had significant decreases of the nocturnal blood pressure reduction percentage; in fact, this measurement was an important indicator reflecting the circadian rhythm of blood pressure. Study results have shown that the nocturnal blood pressure reduction percentage is clearly correlated with OSAHS. Therefore, we considered that moderate and severe OSAHS would influence the circadian rhythm of blood pressure; in addition, the incidence of clinical endpoint events among hypertension patients with OSAHS was higher than that of general hypertension patients.

The main pathological feature of OSAHS is intermittent hypoxia. During sleep, the recurrent hypoxemia and hypercapnia in OSAHS patients can stimulate the central nervous system and the peripheral chemoreceptors, which already have increased sensitivity, to increase the activity of the sympathetic nervous system, thus increasing blood pressure. In addition, repeated awakening can further stimulate sympathetic nerve excitation to increase plasma levels of catecholamine, renin, and angiotensin II, thus causing peripheral blood vessel constriction and eventually causing an increase in nocturnal blood pressure.

In addition to its influence on the sympathetic nervous system, hypoxia can also induce oxidative imbalances in the body. In recent years, scholars proposed the concept that OSAHS was an oxidative stress disorder [6], which meant that it was a pathophysiological process of hypoxia followed by the activation of oxidative stress. Hypoxemia and hypercapnia can produce large amounts of oxygen free radicals in the body, eventually changing the oxidative stress status in the body and inducing pathological inflammatory responses; therefore, the important inflammatory factor component, CRP, is also affected. CRP is mainly synthesized in and secreted by the liver. It is connected by five polypeptide subunits with the same glycosylated structure. Because its levels are significantly increased in hypertension patients, it is currently regarded as an independent risk factor for hypertension [7]. Studies have shown that CRP can aggravate hypertension via several mechanisms. 1) CRP participates in systemic and local inflammatory responses to damage vascular endothelial cells, thus reducing the release of nitric oxide and prostaglandins; additionally, nitric oxide is a powerful vasodilation factor. 2) CRP can promote the thickening of vascular intima to promote the formation and progression of atherosclerosis, thus causing vascular remodeling, increasing resistance, and reducing vascular reactivity to endothelium-dependent vasodilators, eventually resulting in decreases in the blood flow speed during the diastolic period and the mean blood flow speed to aggravate vascular sclerosis and elevate blood pressure [8, 9]. Previous studies have already confirmed that CRP is associated with OSAHS [10]. Our studies also obtained similar results. We suggest that secondary oxidative stress responses resulting from OSAHS-induced intermittent hypoxia constitute an important reason for the elevated serum CRP levels in OSAHS patients and that elevated CRP will further influence blood pressure to cause an abnormal circadian rhythm of blood pressure.

### Conclusions

In summary, intermittent hypoxia induced by OSAHS increases blood pressure through its influences on the sympathetic nervous system. In addition, OSAHS also influences the oxidative stress balance and increases inflammatory factor CRP levels in the body to further change the circadian rhythm of blood pressure. Therefore, an effective improvement of OSAHS has important significance in the control of blood pressure within the standard range.
